# Oteseconazole (VT-1161) for the Management of Dermatophyte Toenail Onychomycosis: A Review of Phase II Trial Subgroup Efficacy and Extended Follow-Up Safety

**DOI:** 10.3390/pathogens15101098

**Published:** 2026-10-21

**Authors:** Aditya K. Gupta, Elizabeth A. Cooper

**Affiliations:** 1 Division of Dermatology, Department of Medicine, Temerty Faculty of Medicine, University of Toronto, Toronto, ON M5S 1A8, Canada; 2 Independent Researcher, London, ON N5X 2P1, Canada

**Keywords:** onychomycosis, azole, oral antifungal, oteseconazole, clinical trial, antifungal resistance

## Abstract

Oteseconazole, a synthetic tetrazole antifungal, provides pharmacological advantages that suggest it can be a safe and effective treatment for *Trichophyton rubrum* onychomycosis. A Phase II study of oteseconazole (VT-1161) for onychomycosis demonstrated good efficacy and safety up to Week 60 in the dosing regimens tested: 300 mg for 12 weeks; 300 mg for 24 weeks; 600 mg for 12 weeks and 600 mg for 24 weeks (all given once daily for 2 weeks, then once weekly for remaining weeks). Additional analysis of efficacy was performed using patient factors that can impact onychomycosis. Similar efficacy rates were found across all four regimens tested. Patient factors (<50 years of age, <50% affected nail area) were more strongly associated with improved outcomes than dose escalation of oteseconazole but did not necessarily predict better overall outcomes. No significant safety concerns were found up to Week 60. New extended follow-up data from Week 60 to Week 96 demonstrated long-term plasma retention of oteseconazole with no additional oteseconazole-related adverse events. Accordingly, clinical photographic assessment at Weeks 72 and 96 showed improvement in the target toenail; however, the number of evaluable patients was limited (*n* = 22), and mycology testing was not performed. The tetrazole structure of oteseconazole significantly reduces the risk of drug–drug interaction compared to traditional azoles (itraconazole, fluconazole). The favorable safety and efficacy profile suggests oteseconazole could be a potential alternative to itraconazole for onychomycosis at any of the tested doses.

## 1. Introduction

Oral terbinafine is considered the gold standard for dermatophyte onychomycosis therapy and has been a primary choice for over 20 years. However, the recent development of dermatophyte resistance to terbinafine therapy is prompting a search for alternatives to oral terbinafine where resistance is suspected [1]. Oral azoles such as itraconazole and fluconazole have a significant history of use for the treatment of dermatophytosis, but they have lower efficacy, and the risk for drug interactions may deter their use in many onychomycosis patients.

As an azole antifungal, oteseconazole has potential activity against superficial dermatophyte fungi in addition to its US FDA-approved indication, recurrent vulvovaginal *Candida* species [2,3,4]. In contrast to the standard imidazole and triazole antifungal agents, oteseconazole is a tetrazole. The tetrazole structure has significantly lower affinity for human CYP enzymes, which greatly reduces the potential for drug–drug interactions when compared with gold-standard azoles (for example, itraconazole) [2]. Reduced risk of drug–drug interactions is a significant advantage for an antifungal, particularly for onychomycosis, where affected patients tend to be older and present with a polypharmacy profile.

Oral oteseconazole also exhibits an exceptionally long median terminal plasma half-life of 138 days versus oral terbinafine (16.5 days) [5] or oral itraconazole (34–42 h ≤ 2 days) [6]. The long half-life may provide an extended duration of antifungal efficacy and allow for reduced frequency and/or duration of dosing compared to other oral antifungals, which may aid patient compliance. Several oteseconazole dosing regimens have been evaluated for dermatophyte toenail onychomycosis and have demonstrated good efficacy and safety over a 60-week Phase II clinical trial [7].

To support future consideration of oteseconazole use for onychomycosis, we present a review of demographic subgroup efficacy from a Phase II trial, with oteseconazole dose selection as a focus. Additionally, we provide details of the favorable pharmacokinetic (PK) profile and safety profile of oteseconazole dosing over an extended follow-up to Week 96, which support further investigation of oteseconazole as an alternative *Trichophyton (T.) rubrum* onychomycosis therapy.

## 2. Methods

**Figure 1:**
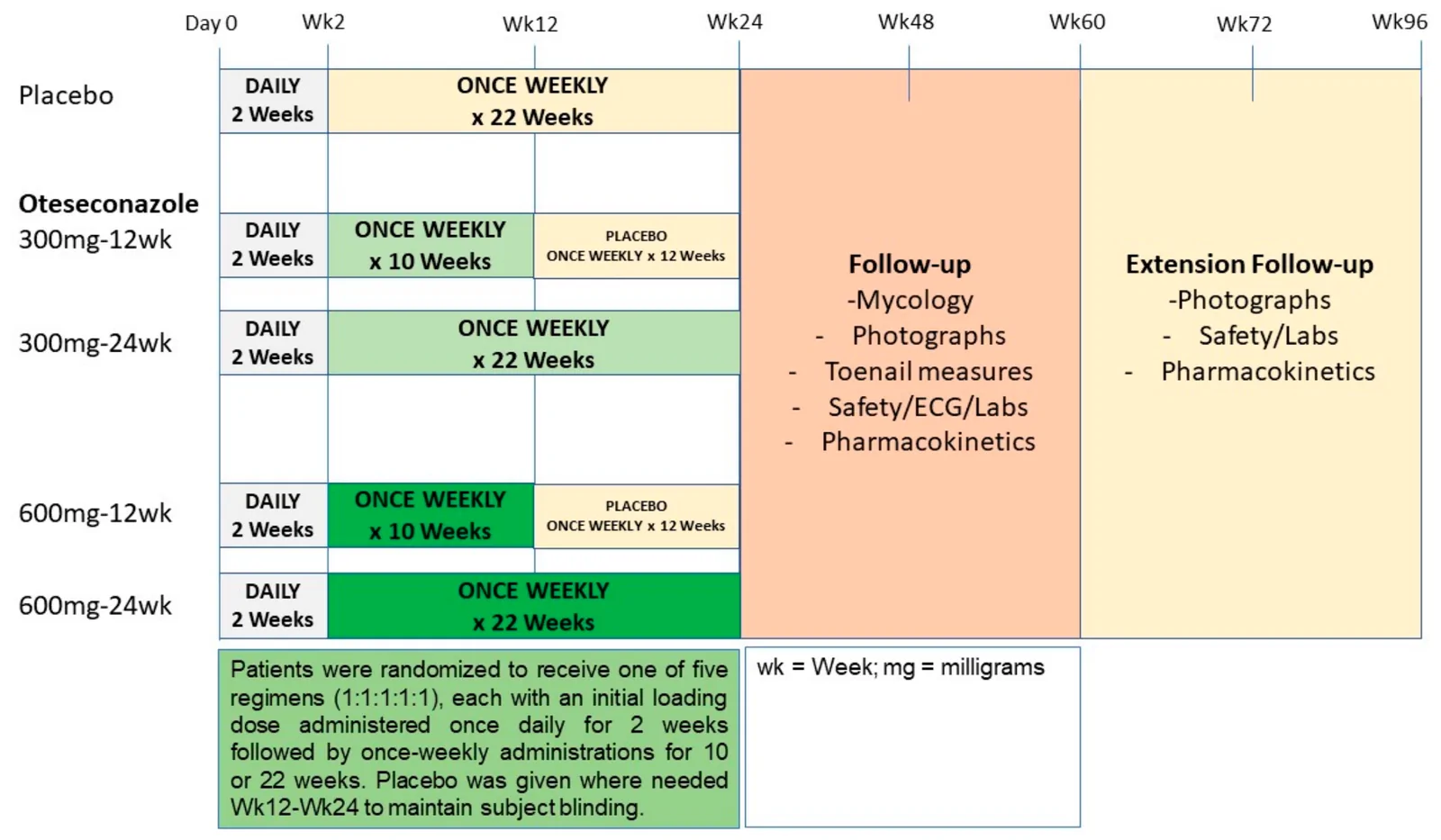
Oteseconazole Phase II study dosing schematic and summary of assessments during the follow-up/extension follow-up periods [7].

A Phase II, multicenter, randomized, double-blind, placebo-controlled, parallel-group, dose-ranging study of oteseconazole (VT-1161; ClinicalTrials.gov identifier NCT02267356) was performed with follow-up to Week 60, as reported in a prior publication from 2021 [7]. The Phase II study received ethics board approval, and written informed consent was obtained from all subjects for study participation, including publication and re-disclosure of medical record information (Chesapeake Institutional Review Board, protocol VMT-VT-1161-CL-005; ethics approval number Pro00010567). All subjects were confirmed to have dermatophyte onychomycosis in at least one great toenail by microscopy and culture prior to enrollment and randomization to a dosing arm. The 5 study dosing arms were: 300 mg for 12 weeks (300 mg-12 wk), 300 mg for 24 weeks (300 mg-24 wk), 600 mg for 12 weeks (600 mg-12 wk), 600 mg for 24 weeks (600 mg-24 wk), and placebo (Figure 1). Dosing was provided once daily in each arm for the first 2 weeks of the study, then once weekly for the remaining assigned treatment period. Placebo dosing once weekly was provided to the 12-week dosing arms from Week 12 to Week 24 to maintain study dose blinding. Outcomes of the study were initially assessed at Weeks 48 and Week 60; Week 48 corresponds to the most frequently assessed outcome period for oral onychomycosis therapy and was used as the primary outcome timepoint for this analysis.

Patients completing to Week 60 were invited into an extended follow-up period to Week 96 to evaluate additional safety data and PK data for the four oteseconazole dosing regimens (Figure 1). Nail photographs were taken, but mycology testing was not performed. No active treatment was given for onychomycosis during the extension follow-up (Week 60 to Week 96). Week 96 follow-up represents a two-year period of nail outgrowth from the start of the study, which is rarely reported in onychomycosis research.

The study sponsor granted the authors access to the anonymized final data report generated at the end of the Phase II study to provide additional analysis within the scope of the initial oteseconazole research objectives. In 2026, the authors reviewed the existing report data with a focus on efficacy and safety by demographic subgroup categories. The research presented here represents secondary research not requiring further ethics approval, as it relied on data from the anonymized Phase II data report and used only the existing Phase II data with no additional subject interventions or interactions, and there was no potential for re-identification of subjects by the authors.

Analysis focused on the per-protocol (PP) population data of the oteseconazole dosing groups only. Intention-to-Treat (ITT) data were presented previously and confirmed that all 4 oteseconazole regimens provided superior efficacy to placebo for both mycological cure (negative microscopy and negative dermatophyte culture) and complete cure (0% affected area and mycological cure) [7]. The ITT population analysis, as a real-world estimate, used a last observation carried forward method to provide a conservative estimate of efficacy for all randomized patients; it was pursued regardless of compliance with the regimen and follow-up visits completed and thus underestimated efficacy. Though PP analysis may overestimate real-world efficacy, both approaches are needed to fully assess drug efficacy. We seek here the most accurate cause-and-effect data possible for patients receiving oteseconazole doses as intended per the protocol in order to draw optimum conclusions about the data regimens. ITT patients who do not complete dosing or do not return for follow-up cannot provide this information and will bias collection of accurate dosing efficacy/safety information. A PP population provides better potential for more explanatory evaluation of dosing and subgroup effects compared to an ITT population [8].

### 2.1. Treatment Efficacy

Treatment efficacy was reviewed for the target great toenail up to Week 60. Mycological cure (as previously defined) was a primary outcome. Clinical cure was also a primary outcome, defined as 0% target toenail involvement. We focused on clinical cure for this PP analysis, as mycological cure in the published study was high at all timepoints [7], and variations in cure rate may be better observed using nail clearance alone. Additional demographic subgroup clinical efficacy to Week 60 was assessed for patient age, sex, baseline area involvement (%) and Body Mass Index (BMI).

Statistical testing of cure rate distribution across oteseconazole dosing groups was performed using Chi-square testing and 95% confidence intervals. The non-parametric McNemar’s test was used to compare paired cure rate proportions from Week 48 to Week 60 (two-tailed, 95% confidence interval).

In the extension follow-up to Week 96, target toenail photographs were taken, but affected area measurements and mycology sampling were not pursued, as the extension was performed for safety review and pharmacokinetic data only. Where target toenail photographs were available for Weeks 72 and 96, the authors performed a visual assessment of serial patient photographs from Week 48 to Week 96. Clinicians visually determine quantitative nail area measures in clinical trials as routine practice. Photograph review provides a suitable proxy for clinical evaluation and is also a routine procedure in onychomycosis clinical trials. Visual analysis is further facilitated by the high quality and standardization of photographs taken in the study. Although mycology testing is essential for a complete analysis of cure, the literature rarely provides a clear nail that allows positive microscopy or culture, especially in an immunocompetent individual (though not unknown). Visual clearance therefore remains a proxy for cure, despite limitations.

The clinical cure status at Week 48 was classified as Cure (clinical cure, 0% nail involvement by visual analysis of photos) or No Cure (any clinical involvement > 0%). Nail involvement was then assessed visually across the photos for Weeks 60, 72 and 96 in comparison to the Week 48 photo, with grading relative to Week 48 defined as follows: Cure (New Cure or No Relapse of Cure) or No Cure (Improved, Minimal Change or Worsening).

### 2.2. Safety Evaluation

The safety endpoints included the number of ITT subjects who experienced treatment-emergent adverse events (AEs) and laboratory abnormalities. Severity and causal relationship to the study drug were determined by the reporting site investigator. Safety data was collected in all weeks of the trial up to Week 96.

Any recorded AE that occurred after initiation of the study drug was considered treatment-emergent and recorded by terms corresponding to the Medical Dictionary for Regulatory Activities (MedDRA) Version 18.0 by preferred term and system organ classification (SOC). All events were assessed by the investigators for relation to the study treatment (unrelated, unlikely related, possibly related or related). An AE was further classified as a Serious Adverse Event (SAE) where the event was: death; life-threatening; requires inpatient hospitalization or prolongation of existing hospitalization; results in a persistent or significant disability/incapacity; or results in a congenital anomaly or birth defect.

The frequencies of any AEs and SAEs that were possibly or probably related to oteseconazole were tabulated across the dosing groups for review of relative safety with each regimen, both in the initial study phase to Week 60 and during the long-term follow-up up to Week 96.

### 2.3. PK Evaluation

Plasma PK analysis was performed in subjects who were assigned to an oteseconazole treatment arm, beginning at Week 2 after completion of the daily dosing phase. Plasma PK collection continued at Weeks 4, 8, 12, 18 and 24, then onward at 12-week intervals until follow-up completion. PK parameters for the oteseconazole dosing groups were calculated by the study sponsor using Phoenix^®^ WinNonlin^®^ Version 6.2 or later, with statistical analysis performed in SAS^®^.

## 3. Results

The study was performed from 2014 to 2017. Of the 259 subjects originally randomized in the study, 212 patients were given oteseconazole dosing (ITT population). Per-protocol oteseconazole dosing was completed by 168 patients to Week 60 (Table 1). The extension follow-up to Week 96 included 132 patients. The majority of patients were White and male, with onychomycosis for 5 years or more (Table 1). The infecting organism for the majority was found to be *T. rubrum* (98%) by standard culture methods. Patient characteristics were similar across oteseconazole treatment groups.

**Figure 2:**
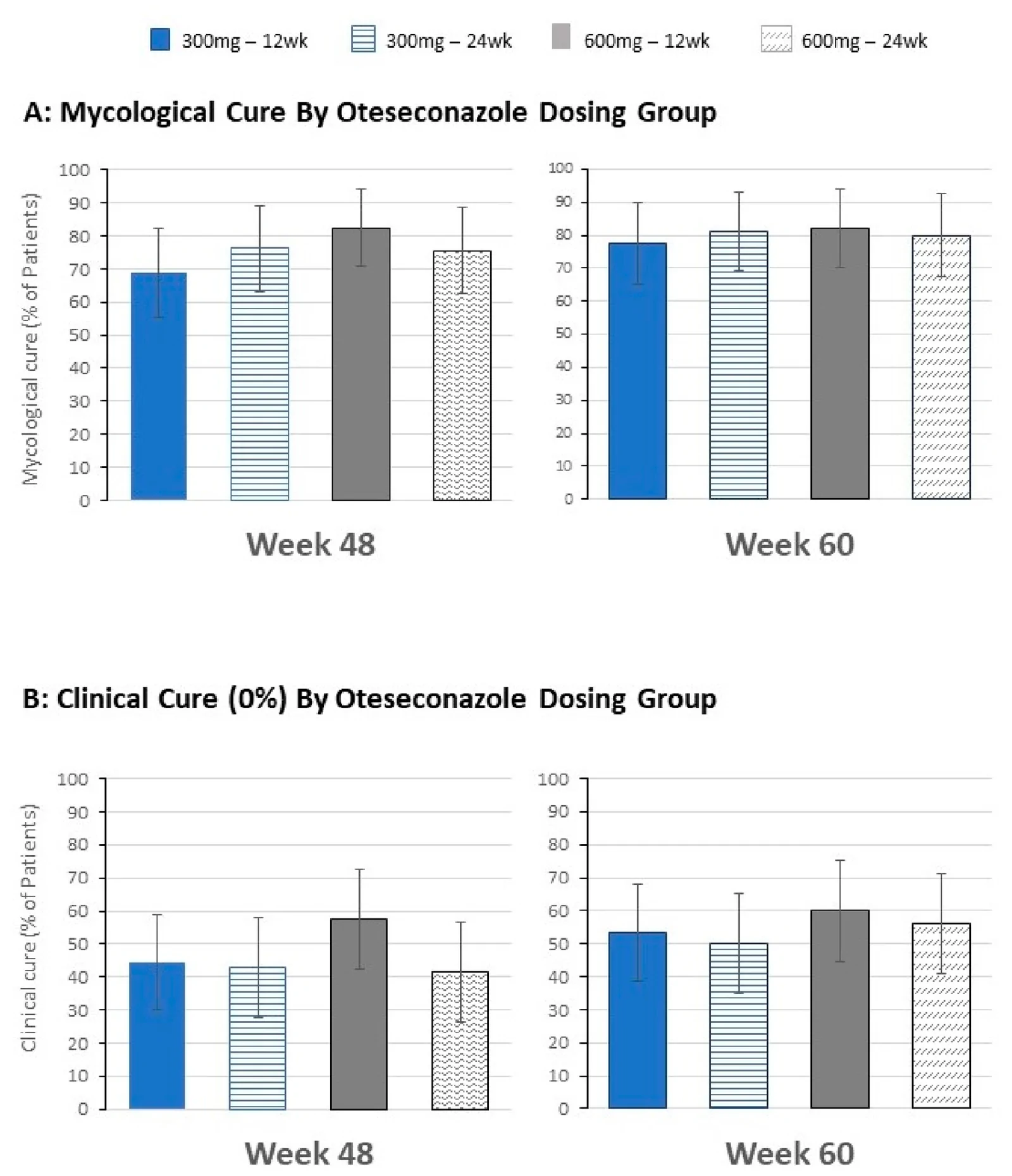
Per-protocol cure rates at Week 48 and Week 60 for the oteseconazole dosing groups.

All oteseconazole dosing groups showed good rates of mycological cure at Week 48, with no significant difference found between groups, and remained high to Week 60 (Figure 2A). Though clinical cure for the 600 mg-12 wk oteseconazole was numerically higher than the other three oteseconazole regimens, the difference did not reach significance, and clinical cure rates were similar across all oteseconazole dosing groups by Week 60 (Figure 2B). Clinical cure rates were much lower than mycological cure rates for all four oteseconazole regimens, suggesting that patients were not able to fully grow out target toenail involvement by Week 48. In other words, whilst there may be mycological cure (as per negative microscopy and culture), the diseased nail grows out slowly and often takes longer than 48 weeks to show complete clinical clearing, in part, depending on the extent of diseased nail at baseline.

### 3.1. Demographic Subgroup Efficacy

**Figure 3:**
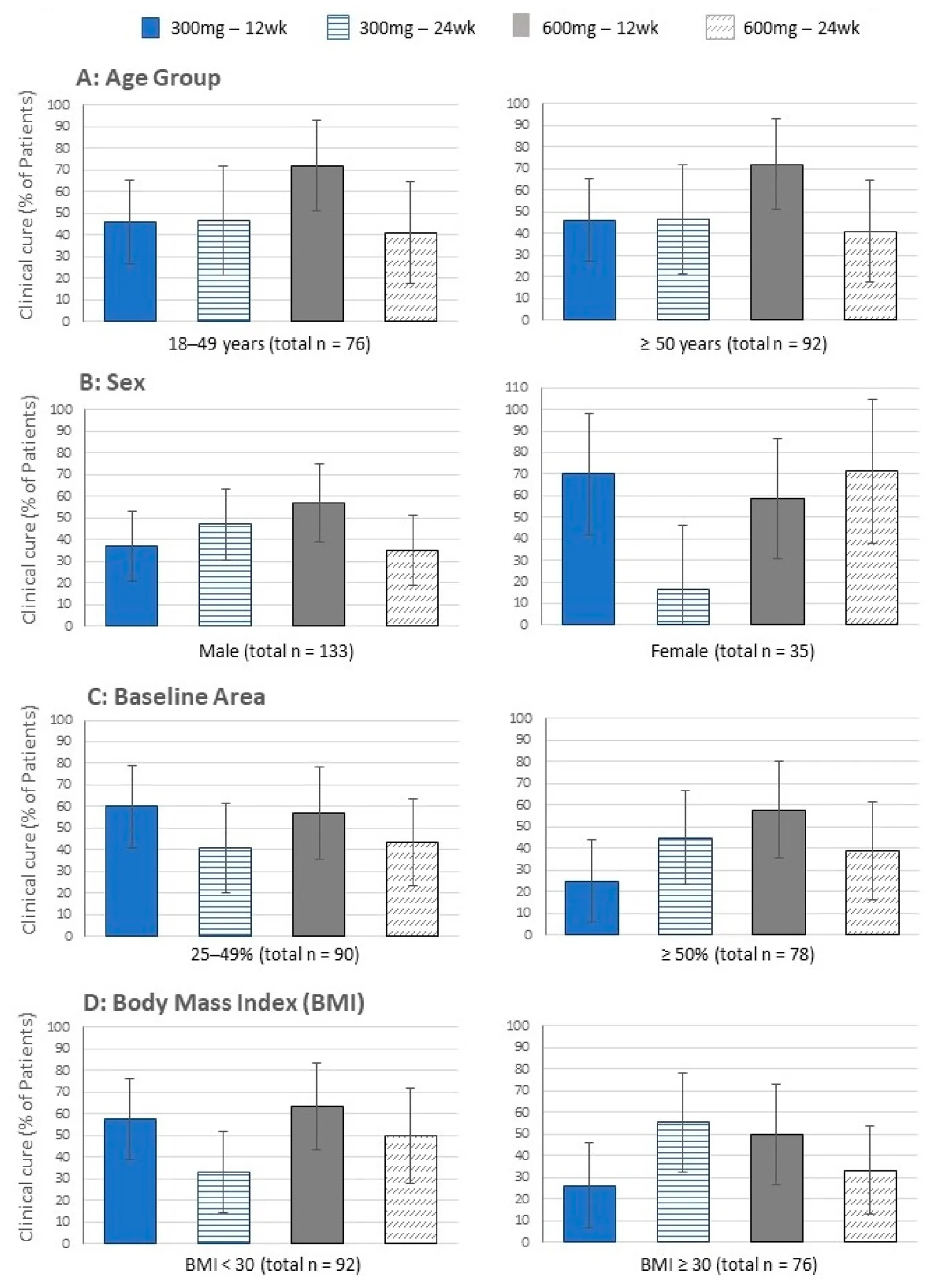
Per-protocol Week 48 clinical cure (0%) rates by oteseconazole dose and demographic subgroups.

Clinical cure rates at Week 48 varied widely across dose groups for most of the reviewed subgroups (Figure 3A–D), but no significant difference was found across the dosing groups. Wide clinical cure disparities in the sex analysis (Figure 3B) result from the small number of female patients available (particularly in the 300 mg-24 wk dosing group) and likely do not reflect a true subgroup effect.

**Figure 4:**
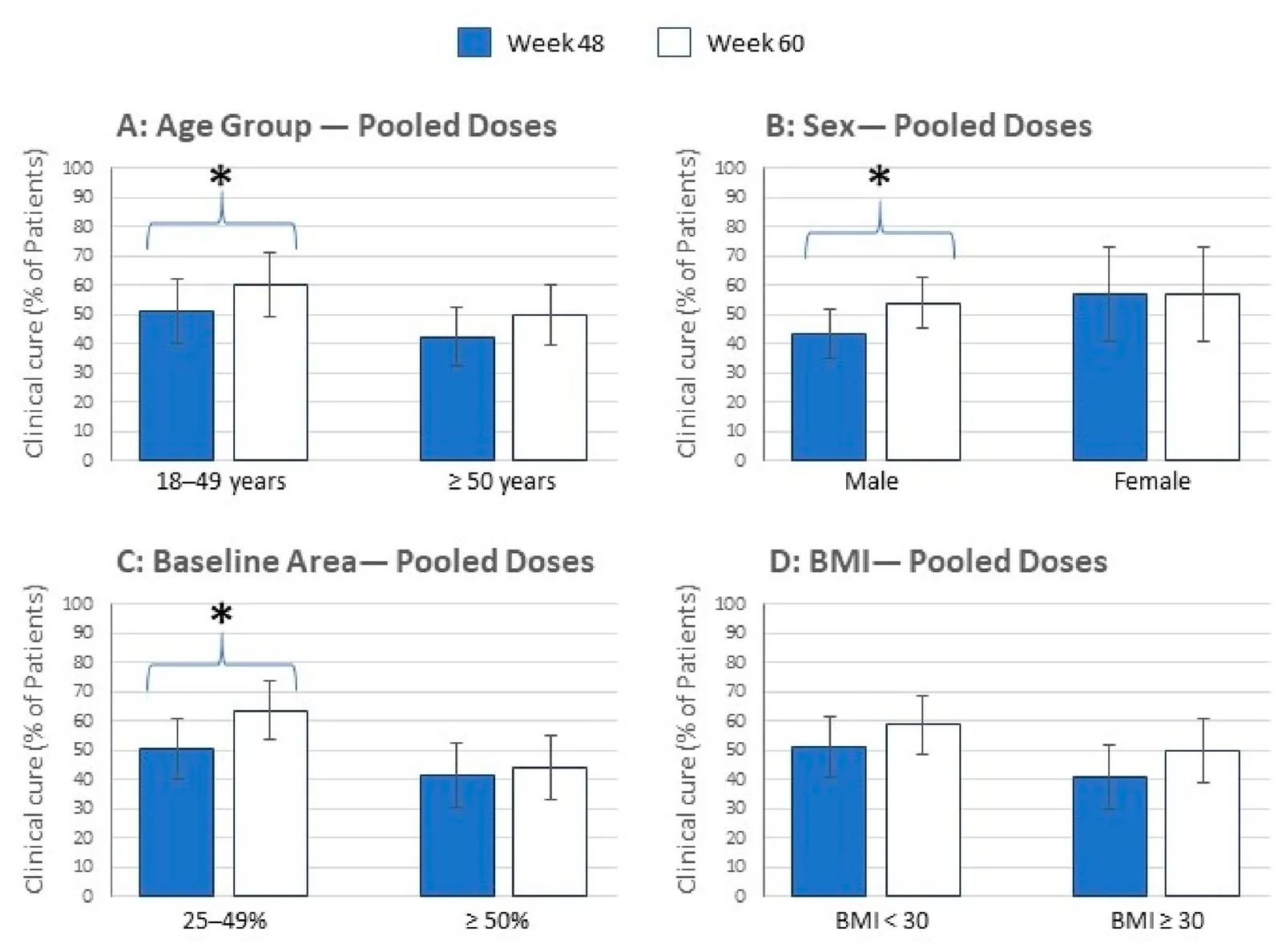
Per-protocol clinical cure (0%) rates, pooled for all oteseconazole dose regimens, compared between Week 48 and Week 60 for each demographic subgroup. * = Week 60 cure rate significantly different from Week 48 cure rate (*p* < 0.05) for the reviewed subgroup based on McNemar’s test, which was used to analyze the significance (two-tailed) of the difference between pairs of correlated proportions.

As dosing differences did not seem to affect cure rates significantly, possibly because all regimens achieved similar profiles showing high systemic absorption (discussed in greater detail in Section 3.3), further analysis was done using pooled dosing to provide increased subgroup sizes for further comparison of Week 48 and Week 60 clinical cure rates with the patient factors (Figure 4A–D). Clinical cure was significantly different (*p* < 0.05) from Week 48 to Week 60 in the 18–49-year age group (Figure 4A), in males (Figure 4B) and in the 25–49% baseline area (Figure 4C). The data suggests that these patient factor groups may be relevant in achieving increased improvements from Week 48 to Week 60. Age and nail severity are well-known factors affecting cure potential in other onychomycosis studies. Younger age may reflect better health status and more active nail growth, which increases the chance of achieving clinical cure. Lower area of involvement, as a proxy for less severe infection, may need less time to fully grow out and has a higher chance of achieving outgrowth within the follow-up period than more severely affected nails. These factors are not likely to be independent of each other (i.e., younger patients tend to have less severe infection and faster nail growth), but this was not tested statistically. Although only male patients showed significant change in clinical cure to Week 60, the small number of females assessed appeared to develop clinical cure sooner and maintain cure to Week 60; statistical significance may not represent clinical significance for sex grouping. Statistical testing also does not confirm a significant difference between patient factor groups at Week 60 (i.e., <50 yrs cure rate not significantly higher than ≥50 yrs at Week 60), and thus the assessed patient factors alone do not necessarily predict better overall outcomes with oteseconazole dosing.

### 3.2. Safety Results

Overall, up to Week 60, the four regimens of oteseconazole were well tolerated. A tabulation of all AEs regardless of relation to study treatment showed that AE profiles of the oteseconazole regimens were similar to those of placebo, as published in the initial data review [7]. Most AEs occurred during the first 12 weeks of treatment. The majority of these AEs were mild to moderate and not related to the study treatment. AEs were ‘possibly related’ or ‘related’ to the study treatment per physician assessment in only 19 total ITT patients to Week 60, at rates of 6% to 11% across the treatment groups (Table 2). Most of the related AEs were gastrointestinal or nervous system events, with nausea and dysgeusia being the most frequently observed events in those categories, respectively. Oteseconazole 600 mg-24 wk treatment had the highest frequency of AEs with possible relation to treatment and the most treatment discontinuations due to an AE. No SAEs to Week 60 were related to study drug treatment, and there were no deaths to Week 60.

Mean values for vital signs were generally stable over time and similar between the oteseconazole treatment groups and placebo. During the study, no subject had abnormal ECG findings that were clinically significant. No changes in hematology, chemistry or urinalysis values were associated with oteseconazole use. Chemistry analytes that demonstrated on-treatment shifts to Grade 3 or 4 were mainly liver function tests (LFTs), including alanine aminotransferase (ALT), aspartate aminotransferase (AST), and total and direct bilirubin. These occurred in less than 1% overall or with no more than a single subject per treatment group per visit. None of the observed elevations in LFTs to Grade 3 or 4 were associated with treatment-emergent AEs. There were no signals observed in treatment-emergent elevations in transaminases or bilirubin that clearly suggested hepatotoxic potential of oteseconazole. The number and proportion of subjects with ALT and AST elevations were similar in the placebo and VT-1161 600 mg groups, if not slightly higher in the placebo group, suggesting a lack of dose effect on treatment-emergent elevations.

All patients consenting to enter the extension follow-up were reviewed at Week 72 and Week 96 for any AEs. The subject demographics in the ITT extension population were similar to the baseline demographics of the original study. No AEs with possible or probable relation to oteseconazole were reported in the extension period (Table 2). No SAEs or deaths were reported in follow-up to Week 96. The number of abnormal hematology and serum chemistry results occurring at Week 72 and Week 96 were low, and rates were similar to earlier timepoints in the study and similar between the placebo and oteseconazole groups. The number and proportion of subjects with ALT, AST and total bilirubin elevations were similar in the placebo and oteseconazole groups. None of these elevations were associated with AEs, and none were suggestive of hepatic toxicity by oteseconazole.

### 3.3. Pharmacokinetics

**Figure 5:**
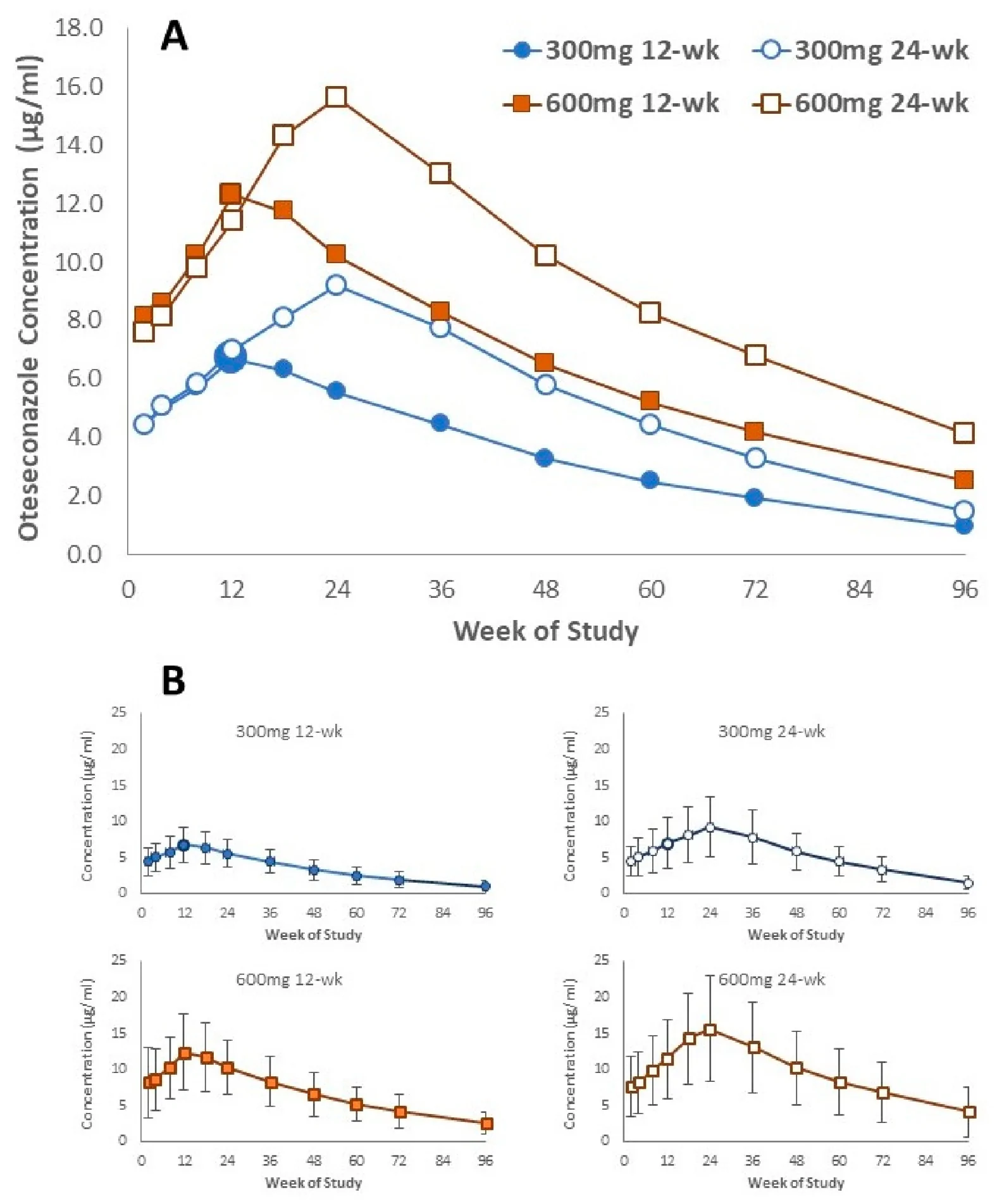
Comparative oteseconazole plasma concentrations ((**A**): µg/mL, arithmetic mean) per dose group and study timepoint to Week 96. Concentrations increase with increased dose and increased duration of oteseconazole use. Plasma levels continue to increase with ongoing dosing, then gradually reduce during post-treatment follow-up. Oteseconazole remains detectable through to Week 96 of the extended follow-up. Individual regimen data is presented in (**B**), showing time course of oteseconazole plasma concentrations with standard deviations of the arithmetic mean added to document variability in the mean values in each group.

Plasma concentrations of oteseconazole increased from the end of daily use at Week 2 to the end of dosing at Week 12 (300 mg-12 wk; 600 mg-12 wk) or Week 24 (300 mg-24 wk; 600 mg-24 wk). After the end of dosing, oteseconazole mean plasma concentrations gradually decreased over time but remained above 2 µg/mL for all four oteseconazole regimens to Week 60 (Figure 5). Oteseconazole mean plasma exposures continued to decrease during the extension follow-up, with residual values at Week 96 ranging from 0.935 µg/mL in the 300 mg-12 wk treatment arm to 4.11 µg/mL in the 600 mg-24 wk treatment arm (Figure 5).

Mean oteseconazole plasma half-life (t_1/2_) values for the four treatment arms ranged from 28.5 weeks in the 300 mg-12 wk treatment arm to 43.3 weeks in the 600 mg-24 wk treatment arm. While there was an apparent increase in oteseconazole plasma t_1/2_ values with increasing dose and/or duration in this study, it was not clear based on a population PK model that there was a dose and/or duration dependence on oteseconazole t_1/2_. Per the safety data evaluated here to Week 96, no long-term safety issues associated with oteseconazole retention were detected. It cannot be ruled out that other safety issues may arise during the retention of oteseconazole; more long-term observation is needed.

While the plasma PK data is favorable, the data represents mean concentrations and therefore any individual patient may not achieve mean plasma levels. PK levels were not assessed in relation to clinical outcomes here and may not correlate with mycological or clinical cure rates. In particular, to be effective, oteseconazole must reach not only plasma but the nail bed, subungual keratin, and the ventral or intermediate nail plate. Like itraconazole and terbinafine, plasma distribution of oteseconazole is followed by rapid distribution into toenails, as reported in a small number of tested patients. The nail data is not presented, as the nail PK population is not representative of the plasma population or PP cure rate population reported in this paper. Wider investigation of oteseconazole nail penetration and retention is needed, particularly in the context of mycological activity and assessing the potential for dermatophyte resistance to oteseconazole during a long retention period.

### 3.4. Extension Clinical Efficacy

In the PP extension population, 22 patients had photos available for an exploratory visual review of extension follow-up efficacy status as follows: 13 patients to Week 72 (300 mg-12 wk-1 pt; 300 mg-24 wk-2 pts; 600 mg-12 wk-7 pts; 600 mg-24 wk-3 pts) and nine patients to Week 96 (300 mg-12 wk-5 pts; 300 mg-24 wk-3 pts; 600 mg-12 wk-1 pt; 600 mg-24 wk-0 pt). As the distribution of follow-up patients was unbalanced across the treatment groups, visual review data was pooled for all subjects for simplicity of data presentation.

**Figure 6:**
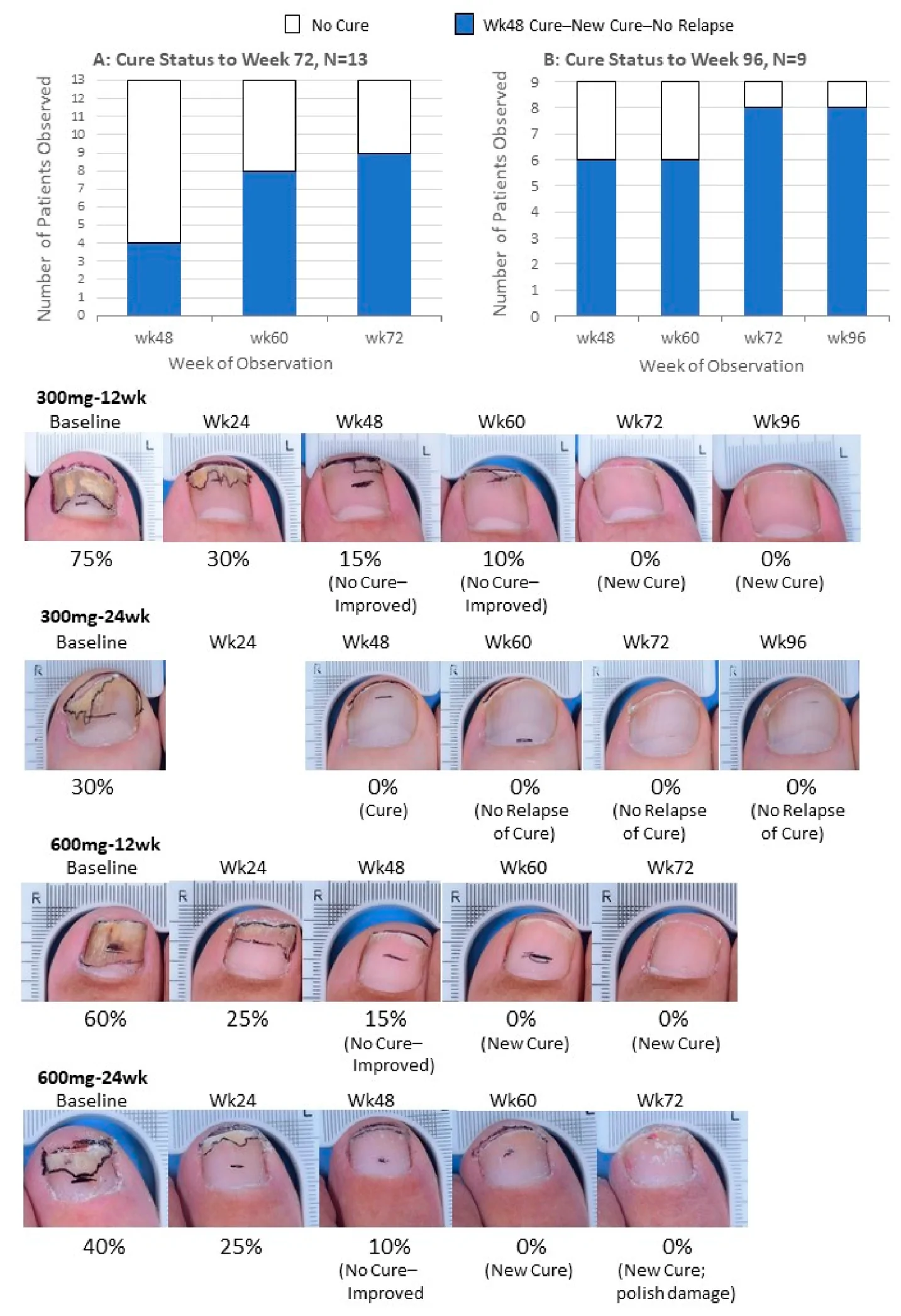
Extension population clinical cure status observed by clinic photos at Week 72 or Week 96. For Weeks 60, 72 and 96, cure status is defined as follows: Cure (New Cure or No Relapse of Cure) or No Cure (Improved, Minimal Change or Worsening). Subject photos provide representative clinical progressions of onychomycosis and author cure grading labels by visual assessment across the long-term observation period. In the small observed sample (*n* = 22), clinical clearance rates by visual assessment increased from Week 48 to the extension follow-up, and no patients showed signs of clinical relapse based on increasing visual area of the affected target toenail. However, no mycology testing was available for correlation with clinical cure observations.

Of the 13 patients with photos available to Week 72, four (30.8%) showed clinical cure (0% affected area by clinical data provided in report to Week 48; one in 300 mg-12 wk, two in 600 mg-12 wk, and one in 600 mg-24 wk) at Week 48, and their nails appeared to remain visually clear to Week 72 (Figure 6A). An additional four patients achieved clinical cure by Week 60 (0% affected area by clinical data provided in report to Week 60, for two in 600 mg-12 wk and two in 600 mg-24 wk; total cured to Wk60:8/13 = 61.5%), and a final patient showed visual clearance at Week 72 (1 pt in 600 mg-12 wk; total cured to Wk72: 9/13 = 69.2%).

For the nine patients with photos to Week 96, six (66.7%) showed clinical cure at Week 48 (0% affected area by clinical data provided in report at Wk48; three in 300 mg-12 wk, two in 300 mg-24 wk and one in 600 mg-12 wk), which appeared to be maintained through to Week 96 (Figure 6B), and two (8/9 = 88.9%) additional patients achieved visual clearance by Week 72 (two in 300 mg-12 wk), with no appearance of visual relapse at Week 96. None of the patient photos showed evidence of clinical relapse, which was visualized by increasing affected areas after Week 48.

This small sample shows that oteseconazole has some potential to maintain clinical clearance once achieved but has significant limitations. The visual review is not a validated method and is subjective based on the observer. Also, no mycology testing was available to confirm or deny the presence of fungal infection in the nails. Visual clearance with positive microscopy and/or culture has only rarely been reported in the medical literature, but cure classification by visual means only remains speculative. The small sample may not be representative of the long-term outcomes of all trial patients, and too few patients had photos to provide evidence of dose regimen effects on long-term clinical outcomes. A wider study including mycological testing is needed to confirm the long-term potential to cure onychomycosis.

## 4. Discussion

The safety data presented here for oteseconazole confirmed the general safety of a two-week daily loading dose followed by once weekly additional dosing for up to 24 weeks. All doses of oteseconazole were relatively safe for most patients; however, individual patients appeared to experience increased sensitivity to the 600 mg dosing.

Oteseconazole is US FDA-approved to reduce the incidence of recurrent vulvovaginal candidiasis in females who are not of reproductive potential [2]. Oteseconazole is contraindicated in females of child-bearing potential and in pregnant and lactating women based on ocular abnormalities recorded in the offspring of pregnant rats administered oteseconazole. Additionally, its long half-life may present prolonged concern for embryo–fetal toxicity [2]. However, many females with onychomycosis are beyond child-bearing years, and oteseconazole may be feasible for a wider female population with onychomycosis (majority > 45 years old) versus other superficial dermatophyte infections such as tinea pedis/capitis/corporis/cruris (majority < 45 years old) [9]. Clinicians may wish to monitor oteseconazole patients over the long term to detect possible delayed safety effects.

The low affinity of oteseconazole for human cytochrome P-450 enzymes, and the lack of interaction with CYP3A4 in particular, may favor oteseconazole as a possible terbinafine alternative for older onychomycosis patients excluded from using other azoles by their polypharmacy agents. Oteseconazole is an inhibitor of the breast cancer resistance protein (BCRP). Oteseconazole 150 mg once daily for 14 days inhibits BCRP, leading to significant concentration increases in the BCRP substrate rosuvastatin [2,10]. Concomitant use of oteseconazole with BCRP substrates such as rosuvastatin may increase the exposure of the substrate drugs, which may increase the risk of adverse reactions associated with these substrate drugs [2]. The FDA prescribing information for oteseconazole recommends using the lowest possible starting dose of a BCRP substrate or considering a reduction in the dose of substrate drugs and monitoring for adverse reactions [2]. The known list of BCRP substrates includes abrocitinib, deucravacitinib, methotrexate, prazosin, glyburide, metformin, glibenclamide, cimetidine, sulfasalazine, and pantoprazole [10,11,12,13]. When using oteseconazole, the practitioner should be aware of possible drug interactions with the tetrazole.

All tested oteseconazole doses produced high rates of mycological cure (week 48: 68–82%). By comparison to Week 48 pivotal trial data, which used similar design and inclusion/exclusion criteria, terbinafine 250 mg once daily for 12 weeks produced a mycological cure of 70% [5], while itraconazole 200 mg daily for 12 weeks showed a mycological cure of 54% [6]. Per-protocol clinical cure rates for oteseconazole at Week 48 are closely representative of a complete cure rate (0% nail area and mycological cure given the high mycological cure rate seen. The oteseconazole clinical cures ranging from 41–57% at Week 48 appear to exceed the itraconazole FDA pivotal trial complete cure rate (14%) [6] and are comparable to the oral terbinafine FDA pivotal trial complete cure of 38% [5]. In this controlled oteseconazole clinical trial population, the higher and longer oteseconazole dose groups provided no clear advantage over the 300 mg-12 wk group when considering mycological cure and clinical cure at Week 48 and Week 60. Review of the plasma PK data showed similar mean plasma concentration levels across the study period for the 300 mg-24 wk and 600 mg-12 wk dosing groups, which may be a factor in the similar efficacy outcomes noted, particularly within those two treatment groups. It seems that the lowest dosing regimen achieves maximal penetration and effect, perhaps saturating access to the nail bed sufficiently so that additional drug or duration has little additional effect, but this is purely speculative, and our observations are limited by being obtained from a Phase II study in a controlled population rather than a real-world onychomycosis population.

Similarly, we found no advantage of higher or longer dosing in any subgroup analyses based on basic patient factors such as age, sex, baseline area and BMI. Patient factors such as lower baseline area or younger age may have more impact on outcomes in this controlled population than changes in oteseconazole dosing, but they do not necessarily provide increased potential for cure to Week 48 or 60. Future prospectively designed studies are needed to confirm an optimal oteseconazole regimen, considering the impact of additional patient factors that affect normal nail outgrowth such as immunocompromised status, poor perfusion of leg/foot/toes, and foot/toe/gait abnormalities [14,15].

Throughout the follow-up to Week 96, mean plasma concentrations remained above the 0.5 µg/mL minimum inhibitory concentration (MIC) upper limit for dermatophytes [3,4], even with the lowest oteseconazole dose; however, achieving MIC may not correlate with achieving cure, particularly if the doses are not achieved in the nail bed. Prolonged exposure of infecting fungi to oteseconazole in plasma and nails may also present a risk of resistance development if the dosing is not appropriately fungicidal. More work is needed to investigate the risk of long-term oteseconazole exposure for the development of relapse and resistance. More in vitro testing of oteseconazole’s potential to induce resistance in dermatophyte fungi would be beneficial, in addition to prospective clinical trials with extended clinical and mycological follow-up designed to fully investigate long-term resistance potential.

The data from this Phase II trial supports a potential role for oteseconazole as an alternative for onychomycosis when an azole treatment such as itraconazole is indicated. Itraconazole is a primary alternative treatment for onychomycosis that is resistant to oral terbinafine; however, many physicians will not prescribe itraconazole due to potential risks (hepatotoxicity, cardiac dysrhythmia; contraindicated in ventricular dysfunction), as well as an extensive list of potential drug–drug interactions [6]. Other azole alternatives such as voriconazole and posaconazole are discouraged for onychomycosis use in order to conserve utility for systemic fungal infection as long as possible, per antifungal stewardship principles [16]. Oteseconazole could potentially overcome the toxicity concerns and drug–drug interaction problems of many azoles and is currently not labeled for systemic antifungal infections. The prolonged PK profile in plasma and nails may also be beneficial in preventing relapse of onychomycosis versus antifungals with shorter half-lives, but prolonged exposure may also be a factor in dermatophyte resistance development.

There are limitations to keep in mind with this data. In addition to the limitations of a PP analysis versus an ITT analysis, as discussed in the methods, the study is also limited in the Week 60–Week 96 extension phase by a small sample size for observation, with a lack of mycology testing to correlate with visual outcomes; visual outcomes are also subjective as they are based on photographs only, and no validated method of clinical assessment is available. The plasma PK population did not have an identical nail PK population for comparison of oteseconazole levels achieved in plasma versus nails, and the mean PK concentrations were not necessarily achieved by all patients for correlation with clinical outcomes. This analysis presents existing Phase II data as no wider testing has been pursued: the long-term safety profile is excellent in this population, but testing in a wider, less controlled population is required to fully confirm dosing safety. Although oteseconazole shows high clinical cure rates, its outcomes have not been verified against itraconazole in head-to-head trials. Given the need for alternative therapies when terbinafine resistance is seen, we hope to focus more attention on wider testing of this existing drug, which could act as an alternate azole therapy.

The lowest oteseconazole dose regimen, 300 mg-12 wk, appears to provide a good baseline of efficacy and safety, with little apparent gain as dose or duration increased in this trial. Further investigation of dosing in less optimal populations may help assess the need for increased dosing or duration. Longer follow-up observation post-dosing, including mycological testing, is needed to establish complete cure potential and fungal resistance potential associated with the long half-life of oteseconazole. Further investigation of oteseconazole as an alternative for onychomycosis therapy is warranted.

## Figures and Tables

**Table 1:** Oteseconazole Per Protocol Treatment Populations and Demographics.

	300mg-12 wk	300mg-24 wk	600mg-12 wk	600mg-24 wk
**Baseline to Week 60**				
Intention to Treat, Total *N* = 212	53	53	52	54
Per Protocol, Total *N* = 168	45	42	40	41
**Age (years):** Mean Range	49.9 ± 8.29 (36–69)	51.9 ± 9.09 (26–70)	49.7 ± 12.42 (24–69)	49.6 ± 10.51 (26–69)
**Sex**:				
Male Female	35 (78%) 10 (22%)	36 (86%) 6 (14%)	28 (70%) 12 (30%)	34 (83%) 7 (17%)
**Race:**				
White African American American Indian or Alaska Native	39 (87%) 6 (13%)0	40 (95%) 2 (5%)0	34 (85%) 6 (15%)0	32 (78%) 8 (20%) 1 (2%)
**BMI (kg/m^2^):** Mean ± SD Range	29.6 ± 4.10 (21.9–38.2)	29.5 ± 5.38 (19.8–47.5)	31.1 ± 7.84 (21.7–54.6)	31.5 ± 5.44 (22.4–42.5)
**Screening % Nail Involvement:**				
< 50% ≥ 50%	25 (55.6%) 20 (44.4%)	22 (52%) 20 (48%)	21 (52.5%) 19 (47.5%)	23 (56%) 18 (44%)
**Duration of Onychomycosis:**				
< 5 years ≥ 5 years	10 (22%) 35 (78%)	9 (21%) 33 (79%)	7 (18%) 33 (83%)	12 (29%) 29 (71%)
**Dermatophyte Species:**				
*Trichophyton rubrum* *Trichophyton mentagrophytes*	44 (98%) 1 (2%)	41 (98%) 1 (2%)	40 (100%)0	40 (98%) 1 (2%)
**Extension to Week 96**				
ITT, Total *N* = 132	33	37	30	32
PP, Total *N* = 124	32	33	29	30
Photos to Week 72, *n* = 13	1	2	7	3
Photos to Week 96, *n* = 9	5	3	1	0
Total Extension photos for review, *n* = 22	6	5	8	3

**Table 2:** Treatment–emergent Adverse Events (ITT Population) with Relation * to Study Treatment.

**Placebo**	55% of patients reported treatment-emergent AEs—6% were considered related to study treatment		
**(*n* = 47)**	**Related AEs**	**Events Reported (Preferred Terms)**	**Impact of Event on Treatment?**
	1 patient	Abdominal pain (upper), ECG abnormal	No withdrawal of treatment
	2 patients	Dizziness	No withdrawal of treatment
	3/47 (6%)		
**300mg-12 weeks**	55% of patients reported treatment-emergent AEs—6% were considered related to study treatment		
**(*n* = 53)**	**Related AEs**	**Events Reported (Preferred Terms)**	**Impact of Event on Treatment?**
	1 patient	Nausea	No withdrawal of treatment
	1 patient	Constipation	No withdrawal of treatment
	1 patient	Diarrhoea	No withdrawal of treatment
	3/53 (6%)		
**300mg-24 weeks**	49% of patients reported treatment-emergent AEs—6% were considered related to study treatment		
**(*n* = 53)**	**Related AEs**	**Events Reported (Preferred Terms)**	**Impact of Event on Treatment?**
	1 patient	Muscle tightness	No withdrawal of treatment
	1 patient	Onychoclasis	No withdrawal of treatment
	1 patient	Constipation, polyneuropathy	**Treatment discontinued**
	3/53 (6%)		
**600mg-12 weeks**	60% of patients reported treatment-emergent AEs—8% were considered related to study treatment		
**(*n* = 52)**	**Related AEs**	**Events Reported (Preferred Terms)**	**Impact of Event on Treatment?**
	1 patient	Dysgeusia	No withdrawal of treatment
	1 patient	Disturbance of attention, paraesthesia	No withdrawal of treatment
	1 patient	Cough, dyspnoea, muscle spasms	**Treatment discontinued**
	1 patient	Lethargy, tremor, muscular weakness, decreased appetite	**Treatment discontinued**
	4/52 (8%)		
**600mg-24 weeks**	67% of patients reported treatment-emergent AEs—11% were considered related to study treatment		
**(*n* = 54)**	**Related AEs**	**Events Reported (Preferred Terms)**	**Impact of Event on Treatment?**
	1 patient	Dysgeusia	No withdrawal of treatment
	1 patient	Dyspepsia	No withdrawal of treatment
	1 patient	Weight decreases	No withdrawal of treatment
	1 patient	Nausea, decreased appetite	**Treatment discontinued**
	1 patient	Nausea, dysgeusia, autonomic nervous system imbalance, intermittent toothache	**Treatment discontinued**
	1 patient	Nausea, dysgeusia, dizziness, vomiting	**Treatment discontinued**
	6/54 (11%)		
**Other Safety Notes**	No SAEs related/possibly related to treatment and no deaths to Week 60		
**Extension to Week 96**	No related AEs/SAEs or deaths in follow-up Week 60 to Week 96		

* “Possibly related” or “Related” per physician assessment; For non-related AE summary, see previous publication [7].

## Data Availability

The source data report used by the authors for analysis resides with the company currently marketing oteseconazole, Mycovia Pharmaceuticals Inc. The authors were granted confidential online access to the final detailed study report and study photographs in a read-only format by Mycovia; the source data report is not available for public analysis. Secondary research datasets used for this analysis were created by the authors from study report data and photograph review data (not including source photos). The data that support the findings of this study are available on reasonable request from the corresponding author, AKG.
